# Brief report: Older adolescents and young adults may be at higher risk for changes to menstrual cycle length with COVID-19 vaccination

**DOI:** 10.1371/journal.pone.0331346

**Published:** 2025-09-18

**Authors:** Laura A. Payne, Alison Edelman, Blair G. Darney, Eleonora Benhar, Emily R. Boniface

**Affiliations:** 1 Department of Psychiatry, Harvard Medical School, Boston, Massachusetts, United States of America; 2 Division of Women’s Mental Health, McLean Hospital, Belmont, Massachusetts, United States of America; 3 Department of Obstetrics & Gynecology, Oregon Health & Science University, Portland, Oregon, United States of America; 4 OHSU-PSU School of Public Health, Portland, Oregon, United States of America; 5 Natural Cycles Nordic AB, Stockholm, Sweden; University of Melbourne, AUSTRALIA

## Abstract

Existing research has consistently demonstrated that adult women experience a small temporary increase in menstrual cycle length following COVID-19 vaccination; however, less is known about whether these changes differ depending on age. The purpose of this study was to assess differences in menstrual cycle length in older adolescent and adult women following an initial COVID-19 vaccine dose compared to an unvaccinated control group, with all analyses conducted across four separate age groups (18–24, 25–29, 30–34, and 35–45). Participants with menstrual cycles averaging 24–38 days prior to vaccination prospectively tracked menstrual cycles using the fertility awareness application Natural Cycles. We compared the within-individual change in menstrual cycle length pre- to post-vaccination in the vaccination group and across four consecutive menstrual cycles in the unvaccinated group, by age. Results demonstrated a 1.11 day adjusted increase in menstrual cycle length for adolescents and young adults (AYAs; ages 18–24), relative to unvaccinated individuals in that age range, while the three older groups all experienced less than a one-day post-vaccination adjusted increase in cycle length compared to their unvaccinated counterparts. These data show that, while COVID-19 vaccination was associated with longer menstrual cycle length for all groups overall, greater increases in menstrual cycle length were observed in AYAs. This suggests that younger individuals are more susceptible to menstrual cycle changes following COVID-19 vaccination.

## Introduction

Existing research has shown that COVID-19 vaccination is associated with changes in menstrual cycle length in adult women, with most studies revealing a small, temporary increase in menstrual cycle length [e.g., 1,2–7] (for review, see [8]). However, less is known about how these changes may differ in younger populations, with one retrospective study finding self-reports of both longer and shorter cycles in adolescent girls ages 12–15 [9]. A recently published prospective study of adolescents who received the COVID-19 booster vaccine found that the girls who received the booster experienced shorter cycles post-vaccine compared to a group that did not receive the booster during the study [10]. This suggests the possibility that younger populations may experience greater variability of menstrual cycle changes post-vaccination compared to adults.

Mechanisms resulting in menstrual cycle abnormalities resulting from COVID-19 vaccination are not known. However, a large body of research suggests that the hypothalamic-pituitary-ovarian axis (HPO-axis) interacts directly with immune and inflammatory processes [11–13]. As a result, significant changes in immune function and inflammation triggered by vaccination may directly impact HPO-axis functioning, including estrogen levels, which could impact menstrual cycle characteristics such as menstrual cycle length.

One major limitation of existing research is that studies have failed to analyze within-study data by age, which makes it difficult to draw conclusions about the relationship of COVID-19 vaccination and menstrual cycle changes in different age groups. The purpose of the current study was to evaluate changes in menstrual cycle length following initial vaccination compared to an unvaccinated control group across four age groups (18–24, 25–29, 30–24, 35–45) using a large data set of participants who prospectively tracked menstrual cycles using a fertility awareness app.

## Materials and methods

We conducted a secondary analysis of prospectively collected menstrual cycle data, which were accessed for the purposes of this paper on February 3, 2023. Authors did not have access to any information that could be used to identify individual participants during or after data collection. The initial analysis examined changes in menstrual cycle and menses length following COVID-19 vaccination and has been described in detail elsewhere [4,14]. Briefly, individuals using the digital fertility awareness application Natural Cycles prospectively tracked their menstrual cycle data, consented to the use of their deidentified data for research purposes, and reported the date of their initial COVID-19 vaccination in response to an in-application message. We included menstruating individuals with consecutively tracked cycles before and after vaccination who 1) were aged 18–45 years, 2) reported their vaccination status and date of initial COVID-19 vaccination, 3) had an average menstrual cycle length in the normal range as defined by the International Federation of Gynecology and Obstetrics, i.e., 24–38 days in the three cycles prior to vaccination [15], 4) were at least three cycles post-pregnancy and post-cessation of hormonal contraception, 5) did not identify themselves as menopausal, and 6) had a known country of residence. This study was reviewed and approved by the Oregon Health & Science University Institutional Review Board (Protocol # 00023204), the Natural Cycles research oversight committee, and the Reading Independent Ethics Committee, UK (No 230721).

Each individual contributed four consecutive cycles of data: the three cycles immediately preceding their first COVID-19 vaccination and the cycle in which the vaccine was received. For the unvaccinated cohort, we assigned a notional vaccination cycle from the same time period. Cycles ranged from October 1, 2020 to November 7, 2021; initial COVID-19 vaccines were received between December 13, 2020 and October 31, 2021. We did not assess cycle length changes in the subsequent cycles as almost two-thirds of the sample (62.1%) received a second vaccination dose in the two cycles following initial vaccination and previous work using the same sample identified no significant changes in cycle length in post-vaccination cycles [4].

Our outcome was the within-individual change in cycle length, in days, from the average of the three pre-vaccination cycles to the cycle in which the vaccine was received. Our primary independent variable was an interaction between vaccination status (vaccinated versus unvaccinated) and age at the beginning of the first study cycle (categorized as ages 18–24, 25–29, 30–34, and 35–45 years).

We also included multiple sociodemographic variables collected within the application. Race and ethnicity were self-reported as a single identity: Asian, Black, Hispanic or Latina, Middle Eastern or North African, Native Hawaiian or Pacific Islander, or white. We categorized body mass index (BMI) as underweight, normal weight, overweight, or obese, and country of residence as the United Kingdom or Channel Islands, Continental Europe, United States or Canada, Australia or New Zealand, and some other country. We classified parity, education level, and relationship status as binary variables: nulliparous versus parous, less than a college degree versus college degree or more, and in a relationship (yes or no), respectively. Report of sociodemographic data is voluntary within the application and collection of the variables has changed over time, resulting in a high degree of missing data for some variables. We did not include vaccine brand in our analyses as our previous work has found no association between individual brands and post-vaccination menstrual disturbances [2–4].

### Analysis

We compared all sociodemographic variables between vaccination groups using Pearson’s chi-squared test. We developed both unadjusted and adjusted linear regression models using within-individual change in menstrual cycle length as the outcome and an interaction between vaccination status and age group as the primary independent variable. Adjusted models included race and ethnicity, parity, BMI category, education level, relationship status, and geographic region as covariates, following multiple imputation with chained equations to address covariate missingness. We used the adjusted model to predict the mean change in cycle length (i.e., average marginal effects) for each category of the vaccination status/age group interaction and graphed the estimates and 95% confidence intervals. We then calculated the predicted mean difference between vaccinated and unvaccinated individuals for each age group, i.e., the effect associated with vaccination, based on both the unadjusted and adjusted models. We conducted a sensitivity analysis excluding individuals who received a second dose of vaccine in the same cycle (n = 743) All analyses were conducted using Stata 17.0 (StataCorp, College Station, TX); p-values < 0.05 were considered statistically significant.

## Results

**Fig 1:**
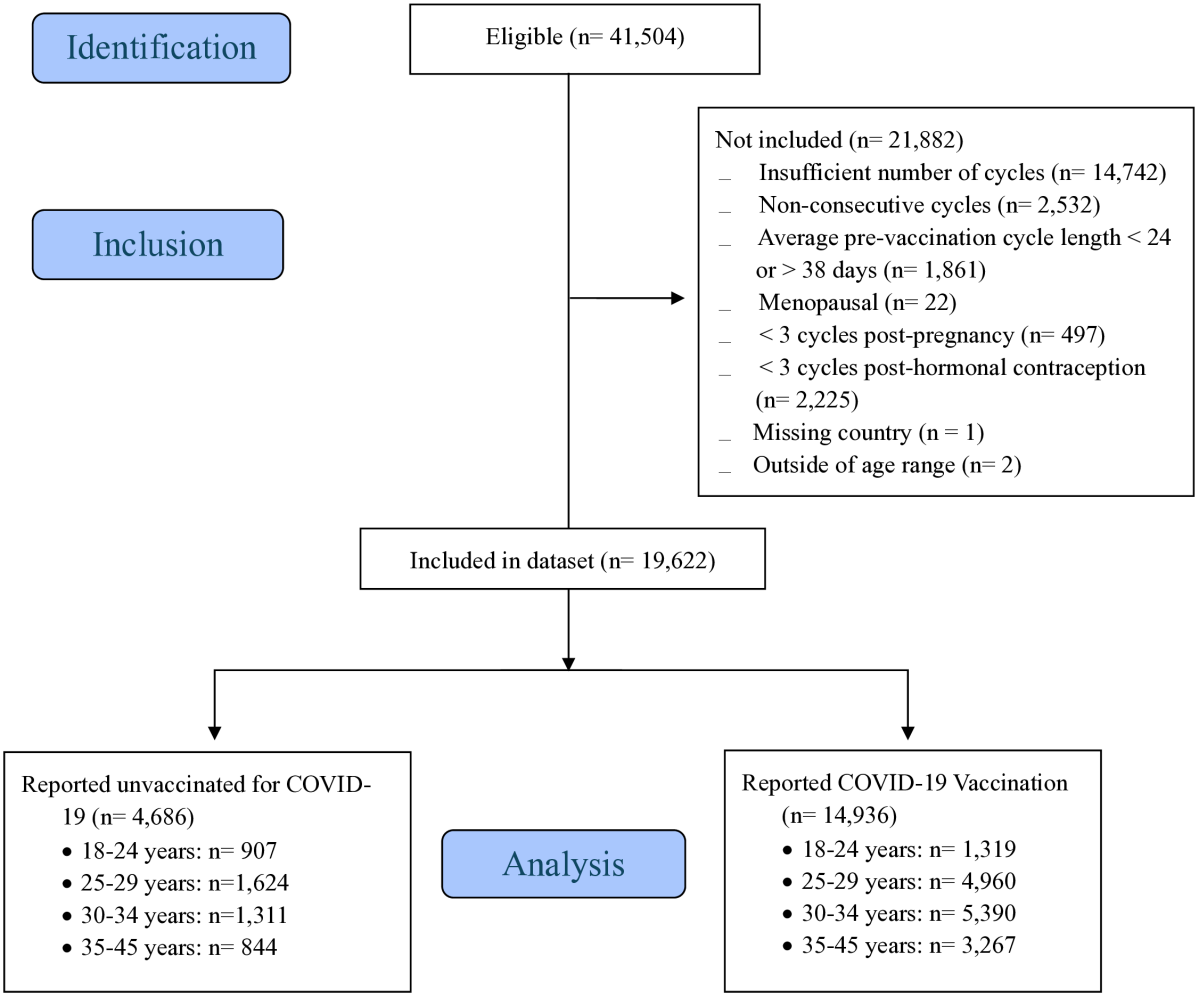
STROBE Flow Diagram.

Our analytic sample included 19,622 individuals out of 41,504 eligible for inclusion (**Fig 1**), representing 78,488 cycles. Vaccinated individuals made up approximately three-quarters of the sample: n = 14,936 vaccinated (76.1%) and n = 4,686 unvaccinated (23.9%). The unvaccinated group was divided roughly equally between the four age groups (**Table 1**): 19.4% (n = 907) ages 18–24, 34.7% (n = 1,624) ages 25–29, 28.0% (n = 1,311) ages 30–34, and 18.0% (n = 844) ages 35–45. The vaccinated group was more likely to be older: 8.8% (n = 1,319) ages 18–24, 33.2% (n = 4,960) ages 25–29, 36.1% (n = 5,390) ages 30–34, and 21.9% (n = 3,267) ages 35–45 (overall p < 0.001). The majority of the sample had at least a college degree (66.4%), was in a relationship (69.1%), and was located in the United Kingdom, continental Europe, the United States, or Canada (93.9%). Unvaccinated individuals were more likely to be nulliparous (68.1% compared to 77.1%, p < 0.001), and live in the United States or Canada (42.7% compared to 24.2%, p < 0.001).

**Table 1:** Characteristics of Study Participants (n = 19,622). Data are n (%).

Characteristic	Unvaccinated (n = 4,686)	Vaccinated (n = 14,936)	Overall (n = 19,622)	p-value
**Age (years)**				< 0.001
18-24	907 (19.4)	1,319 (8.8)	2,226 (11.3)	
25-29	1,624 (34.7)	4,960 (33.2)	6,584 (33.6)	
30-34	1,311 (28.0)	5,390 (36.1)	6,701 (34.2)	
35-45	844 (18.0)	3,267 (21.9)	4,111 (21.0)	
**Race/Ethnicity**				< 0.001
Asian	20 (0.4)	130 (0.9)	150 (0.8)	
Black	170 (3.6)	346 (2.3)	516 (2.6)	
Hispanic	76 (1.6)	235 (1.6)	311 (1.6)	
Middle Eastern/North African	15 (0.3)	41 (0.3)	56 (0.3)	
Native Hawaiian/Pacific Islander	4 (0.1)	19 (0.1)	23 (0.1)	
White	1,513 (32.3)	5,306 (35.5)	6,819 (34.8)	
No data	2,888 (61.6)	8,859 (59.3)	11,747 (59.9)	
**Parity**				< 0.001
Nulliparous	3,192 (68.1)	11,509 (77.1)	14,701 (74.9)	
Parous	757 (16.2)	1,885 (12.6)	2,642 (13.5)	
No data	737 (15.7)	1,542 (10.3)	2,279 (11.6)	
**BMI (kg/m^2^)**				< 0.001
Underweight (< 18.5)	144 (3.1)	428 (2.9)	572 (2.9)	
Normal weight (18.5–24.9)	2,057 (43.9)	7,274 (48.7)	9,331 (47.6)	
Overweight (25–29.9)	564 (12.0)	1,924 (12.9)	2,488 (12.7)	
Obese (> 30)	233 (5.0)	852 (5.7)	1,085 (5.5)	
No data	1,688 (36.0)	4,458 (29.9)	6,146 (31.3)	
**Education level**				< 0.001
Less than college degree	1,215 (25.9)	2,205 (14.8)	3,420 (17.4)	
College degree or more	2,496 (53.3)	10,540 (70.6)	13,036 (66.4)	
No data	975 (20.8)	2,191 (14.7)	3,166 (16.1)	
**Relationship status**				< 0.001
Not in relationship	631 (13.5)	1,928 (12.9)	2,559 (13.0)	
In relationship	3,002 (64.1)	10,553 (70.7)	13,555 (69.1)	
No data	1,053 (22.5)	2,455 (16.4)	3,508 (17.9)	
**Geographic Region**				< 0.001
UK/Channel Islands	1,098 (23.4)	5,124 (34.3)	6,222 (31.7)	
Continental Europe^a^	1,151 (24.6)	5,433 (36.4)	6,584 (33.6)	
USA/Canada	2,002 (42.7)	3,608 (24.2)	5,610 (28.6)	
Australia/New Zealand	377 (8.1)	390 (2.6)	767 (3.9)	
Other^b^	58 (1.2)	381 (2.6)	439 (2.2)	

^a^56% based in Sweden. ^b^62% based in Brazil.

**Fig 2:**
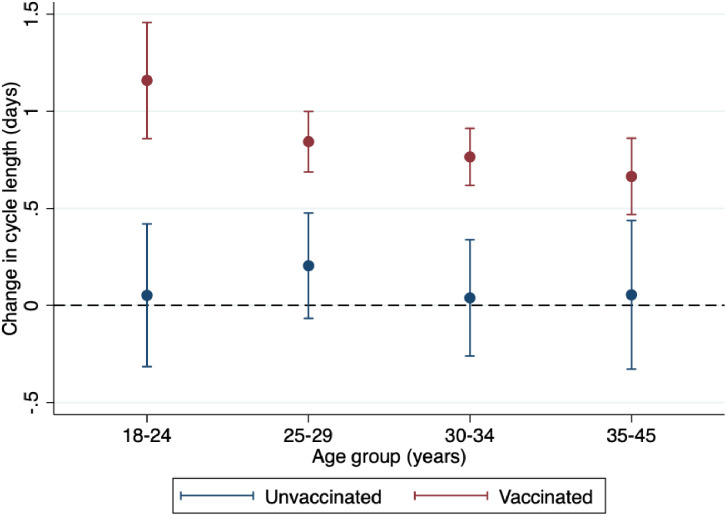
Adjusted within-individual change in menstrual cycle length (in days) from 3 pre-vaccination cycle average to initial vaccination cycle for unvaccinated (blue) and vaccinated (red) individuals by age group. Estimates are adjusted for race and ethnicity, parity, BMI category, education level, relationship status, and geographic region, following multiple imputation with chained equations. Error bars represent 95% confidence intervals; horizontal dashed line indicates no change from the pre-vaccination cycle mean.

When we examined adjusted changes in menstrual cycle length by age group, there were no significant differences for any of the unvaccinated groups (**Fig 2**): 0.05 days (95% CI: −0.32, 0.42 days) for ages 18–24, 0.20 days (95% CI: −0.07, 0.48 days) for ages 25–29, 0.04 days (95% CI: −0.26, 0.34 days) for ages 30–34, and 0.06 days (95% CI: −0.33, 0.44 days) for ages 35–45. In contrast, all vaccinated groups experienced a significant increase in cycle length following vaccination compared to pre-vaccination cycle length. Although the estimates were not statistically different across age groups (p = 0.354), there was a monotonic decreasing trend in post-vaccination changes with increasing age: 1.15 days (95% CI: 0.86, 1.46 days) for ages 18–24, 0.84 days (95% CI: 0.69, 1.00 days) for ages 25–29, 0.76 days (95% CI: 0.62, 0.91 days) for ages 30–34, and 0.66 days (95% CI: 0.47, 0.86 days) for ages 35–45.

The adjusted differences between the vaccinated and unvaccinated group were highest for AYAs; vaccination was associated with a 1.11 day adjusted increase relative to the unvaccinated for individuals ages 18–24 (95% CI: 0.64, 1.57; **Table 2**). The three older groups all experienced less than a one-day post-vaccination adjusted increase in cycle length compared to their unvaccinated counterparts: a 0.64 day increase (95% CI: 0.33, 0.95) for ages 25–29, 0.73 day increase (95% CI: 0.39, 1.06) for ages 30–34, and 0.61 day increase (95% CI: 0.19, 1.03) for ages 35–45. Unadjusted estimates were similar to adjusted estimates across all age groups. Our sensitivity analysis excluding individuals who received two doses of the vaccine in the same cycle produced attenuated estimates within the vaccinated groups, as expected, but trends remained similar (data not shown).

**Table 2:** Predicted mean difference (95% CI) in within-individual change in menstrual cycle length (in days), between vaccinated and unvaccinated cohorts, by age group.

Age group (years)	n	Unadjusted	Adjusted^a^
18-24	2,226	1.12 (0.66, 1.59)	1.11 (0.64, 1.57)
25-29	6,584	0.65 (0.34, 0.95)	0.64 (0.33, 0.95)
30-34	6,701	0.73 (0.40, 1.06)	0.73 (0.39, 1.06)
35-45	4,111	0.63 (0.21, 1.04)	0.61 (0.19, 1.03)

^a^Estimates are adjusted for race and ethnicity, parity, BMI category, education level, relationship status, and geographic region, following multiple imputation with chained equations.

## Discussion

This study evaluated within-individual changes in menstrual cycle length from pre- to post-COVID-19 vaccination across age groups, compared to an unvaccinated control group, using a large sample of AYA and adults who were prospectively tracking menstrual cycles. Results showed significantly longer menstrual cycles post-vaccination in all age groups compared to those in the unvaccinated control group. However, the youngest age group (18–24 years) showed the greatest changes in menstrual cycle length compared to their pre-vaccination averages when contrasted with their unvaccinated counterparts. Even though these findings were not statistically significant, this is consistent with prior research showing small, but significant increases in menstrual cycle length in adults after COVID-19 vaccination [2,4,5], as well as variable [6,9] or shorter [10] menstrual cycle length in adolescents after COVID-19 vaccination. It is possible that the AYA cohort in this group on average did not reflect statistically significant changes because they were slightly older than previous studies focusing exclusively on adolescents. Additionally, the study that found shorter menstrual cycle length following COVID-19 vaccination in adolescents examined only adolescents following receipt of a booster vaccination, which may have impacted menstrual cyclicity differently compared to a first vaccination.

Although the amount of change in menstrual cyclicity (approximately 1 day; on average) may not be of clinical concern, this information allows providers to counsel patients about potential small changes to menstrual cyclicity following vaccination. This information can be a source of reassurance to patients, particularly AYAs, who may find it useful to have the information before vaccination, although previously published studies suggest that menstrual changes do not impact fertility [16,17] or health overall [8].

These data suggest that HPO-axis maturity may be a key factor in determining menstrual cyclicity in response to the COVID-19 vaccine. Younger populations earlier in their reproductive lives may be more susceptible to the impact of the vaccine due to incomplete maturation of the HPO-axis [18,19]. The immune and inflammatory responses following vaccination may therefore have a more significant impact on gonadal hormone signaling, which alters menstrual cycle length [20], thus resulting in longer or shorter menstrual cycles. As individuals age, the HPO-axis becomes more stable and thus less sensitive to significant immune reactions; however, almost no research exists on immune and inflammatory mechanisms of menstrual cycle changes following vaccination so this hypothesis remains speculative. More research exploring these mechanisms is needed.

### Limitations

Limitations to this study include that these data were self-reported using a fertility awareness app, so it is possible that results are not representative of the larger population or may be influenced by self-report or selection bias. However, data were prospectively collected. The data on additional menstrual characteristics (bleeding quantity, flow, past hormonal use, etc.) were also not included in these analyses. Inclusion of these parameters is beyond the scope of this paper, and is not consistently reported by app users, thereby introducing bias and limiting sample size. However, these variables may have a relationship to cycle length that are not accounted for. Similarly, we did not have a sufficiently large sample to assess changes in cycle length by menstrual phase of vaccination within age groups. We also are not able to account for possible confounding variables, such as weight loss/gain, acute illness, or pandemic-related stress or lifestyle changes, that may impact menstrual cycle variability during the study period. Results also may be different in other populations, including those with irregular menstrual cycles, those using hormonal birth control, and those in peri-menopause.

## Conclusions

COVID-19 vaccination was associated with an increase in menstrual cycle length for women, with AYAs experiencing the greatest changes post-vaccination compared to older age groups. Clinicians and policymakers should advise patients and stakeholders of the potential for changes to menstrual cycle length as part of routine education when receiving the COVID-19 vaccination.
